# Identification of Potential Serum Proteomic Biomarkers for Clear Cell Renal Cell Carcinoma

**DOI:** 10.1371/journal.pone.0111364

**Published:** 2014-11-04

**Authors:** Juan Yang, Jin Yang, Yan Gao, Lingyu Zhao, Liying Liu, Yannan Qin, Xiaofei Wang, Tusheng Song, Chen Huang

**Affiliations:** 1 Key Laboratory of Environment and Genes Related to Diseases of the Education Ministry, Department of Genetics and Molecular Biology, Medical School of Xi′an Jiaotong University, Xi′an, China; 2 Department of Medical Oncology, First Affiliated Hospital of Medical School of Xi'an Jiaotong University, Xi′an, China; Deutsches Krebsforschungszentrum, Germany

## Abstract

**Objective:**

To investigate discriminating protein patterns and serum biomarkers between clear cell renal cell carcinoma (ccRCC) patients and healthy controls, as well as between paired pre- and post-operative ccRCC patients.

**Methods:**

We used magnetic bead-based separation followed by matrix-assisted laser desorption ionization (MALDI) time-of-flight (TOF) mass spectrometry (MS) to identify patients with ccRCC. A total of 162 serum samples were analyzed in this study, among which there were 58 serum samples from ccRCC patients, 40 from additional paired pre- and post-operative ccRCC patients (n = 20), and 64 from healthy volunteers as healthy controls. ClinProTools software identified several distinct markers between ccRCC patients and healthy controls, as well as between pre- and post-operative patients.

**Results:**

Patients with ccRCC could be identified with a mean sensitivity of 88.38% and a mean specificity of 91.67%. Of 67 m/z peaks that differed among the ccRCC, healthy controls, pre- and post-operative ccRCC patients, 24 were significantly different (P<0.05). Three candidate peaks, which were upregulated in ccRCC group and showed a tendency to return to healthy control values after surgery, were identified as peptide regions of RNA-binding protein 6 (RBP6), tubulin beta chain (TUBB), and zinc finger protein 3 (ZFP3) with the m/z values of 1466.98, 1618.22, and 5905.23, respectively.

**Conclusion:**

MB-MALDI-TOF-MS method could generate serum peptidome profiles of ccRCC, and provide a new approach to identify potential biomarkers for diagnosis as well as prognosis of this malignancy.

## Introduction

Clear cell renal cell carcinoma (ccRCC) is a renal cortical tumor typically characterized by malignant epithelial cells with a clear cytoplasm and a compact-alveolar (nested) or acinar growth pattern interspersed with intricate, arborizing vasculature. ccRCC represents over 80% of renal cell carcinomas (RCCs) [1], which are the most common form of kidney cancer, accounting for 3% of all cancer diagnoses and more than 100,000 deaths worldwide each year [2]. The most effective treatment for ccRCC is currently surgical resection, partial nephrectomy is considered for tumors smaller than 4 cm in diameter (stage pT1a) and radical nephrectomy for tumors larger than 4 cm [3]. However, ccRCC is associated with a fast rate of extrarenal growth, metastasis (most commonly to the lung, liver, bone or brain) and mortality [1], [4]. The survival rate of ccRCC patients decreases with increasing disease stage [5], [6]. Therefore, the early detection of ccRCC would significantly improve patient diagnosis and outcome.

The identification of biomarkers for the early detection of cancer could lead to the development of efficient treatments, reduce suffering, and lower mortality rates [7]. However, there are currently no biomarkers for the reliable screening of patients with ccRCC [8]–[10]. Human serum contains a complex array of peptides, and some of these could serve as biomarkers because their presence/absence or relative abundance may be correlated with certain diseases and could thus be useful for prognosis or diagnosis [11]–[13]. The identification of differentially expressed peptides and proteins by mass spectrometry (MS) combined with software-generated models capable of discriminating between the spectra of patients with ccRCC and healthy controls could lead to the identification of potential new biomarkers for ccRCC. Here, we report on the use of magnetic bead-based purification approaches coupled with MALDI-TOF MS for the comparative analysis of sera from patients with ccRCC and healthy controls, as well as ccRCC patients who underwent surgical resection. And potential serum biomarkers for detection of ccRCC were then identified by LC-ESI-MS/MS.

## Materials and Methods

### Patients and sample preparation

The study protocol was approved by the Ethics Committee and the Human Research Review Committee of Xi′an Jiaotong University, and each subject has been provided signed informed consent before the work. All samples were collected from the First Affiliated Hospital of Xi′an Jiaotong University between January 1st, 2010 and December 12th, 2011. Clinical data were retrospectively collected from medical record reviews and electronic records, and tumor histology were obtained from pathology. Patients with a known history of other tumors and those with obvious inflammatory diseases were excluded. For clinical variables, age at diagnosis, sex, and tumor stage and Fuhrman grade were considered. Tumor stage was defined according to the seventh edition of the American Joint Committee on Cancer (AJCC) cancer staging manual.

The 64 control serum samples were obtained from healthy donors recruited for this study including 32 men and 32 women with an average age of 51.7 years (range, 31–78 years). Serum samples of ccRCC groups were obtained from 58 ccRCC patients before surgical operation, including 44 men and 14 women with an average age of 54.6 years (range, 33–74 years). Besides, 40 serum samples were obtained from 20 additional paired pre- and post-treatment ccRCC patients, with their post-operative serum samples collected three days after surgery. Clinico-pathological characteristics of all patients were shown in Table 1.

**Table 1:** Clinico-pathological characteristics of 78 clear cell renal cell cancers.

Characters	Number of cases	% of cases
**Age** Median years (range)	58.53 (33–74)	
**Gender**		
Male	58	74
Female	20	26
**Furhman grade**		
G1	6	7.5
G2	54	69
G3	12	15
G4	0	0
unknown	6	7.5
**TNM stage**		
Stage I	48	62
Stage II	30	38
Stage III	0	0
Stage IV	0	0
**T stage**		
T1	48	62
T2	30	38
T3	0	0
T4	0	0
**Lymph node metastasis**		
N0	78	100
N1	0	0
**Distant metastasis**		
M0	78	100
M1	0	0

All blood samples were drawn from non-fasting subjects in a sitting position. The samples were collected in 10 cc serum separator tubes and kept at 4°C for 1 h, then centrifuged at 3000 g for 20 min at 4°C. The serum samples were distributed into 500 µL aliquots and stored at −80°C until use.

### MS analysis: WCX fractionation and MALDI-TOF MS analysis

Samples were separated by magnetic bead-based weak cation-exchange chromatography (MB–WCX) using ClinProt purification reagent sets from Bruker Daltonics. MB-WCX purifications were performed using the Bruker Magnetic Separator according to the manufacturer's protocol. The details of this experiment were reported previously [13]. To prepare the MALDI target, 1 µL of a mixture containing 10 µL of 0.3 g/L α-cyano-4-hydroxy cinnamic acid (HCCA) in 2∶1 ethanol/acetone (volume/volume) and 1 µL of the eluted peptide fraction were spotted onto the MALDI AnchorChip (Bruker Daltonics, Germany) sample target platform (384 spots). To evaluate the reproducibility of the assay, all serum samples were spotted in triplicate. Air-dried targets were measured immediately using a calibrated Autoflex III MALDI-TOF MS (Bruker) with FlexControl software (version 3.0, Bruker) and optimized measuring protocols. Matrix suppression up to 1000 Da, with a mass range of 1000–10,000 Da was set as the default. Instrument calibration parameters were determined using standard peptide and protein mixtures. All measurements were performed in a blinded manner, including patient and control sera in one mixed approach.

Data analyses were performed using the programs Flex analysis v3.0 and ClinProTools v2.2 (Bruker Daltonics, Germany). ClinProTools v2.2 uses a standard data preparation workflow including spectra pretreatment, peak picking, and peak calculation operations, and was applied for the recognition of peptide patterns in this study. For statistical analyses, a k-nearest neighbor genetic algorithm, as implemented in the software suite, was used to identify statistically significant differences in protein peaks among the groups analyzed.

For statistical analyses, three different algorithms of mathematical models were used: Genetic Algorithm (GA), Supervised Neural Network (SNN) and Quick Classifier (QC). The GA algorithm is derived from evolutionary survival in which the best peak clusters are combined into a new feature and the poor clusters are discarded. This process is iteratively repeated until the optimal peak combination is found. The SNN algorithm maximizes the distance of multiple local peak clusters specific to each group. Clusters that provide greater separation are prioritized over those with low separation. Finally, the QC algorithm generates an average spectrum for each group with weighted p-values for each peak. Based on the peak weights, spectra are categorized into either group along with a likeliness value.

### Peptide identification by LC-ESI-MS/MS

After completing the statistical analysis, differentially expressed peptides were identified. The peptide sequences of potential m/z peaks were first determined by LC-ESI-MS/MS, and the identified sequence data were then subjected to a Mascot database search to identify the corresponding full-length protein matches.

### Statistical Analysis

Statistical analyses were conducted using GraphPad Prism v5.0 (GraphPad Software). Correlations between the mean expressions of all detected m/z peaks with Clinicopathological characteristics of ccRCC patients were evaluated using the Spearman rank-order correlation coefficient. All data were expressed as the mean ± SD. The results were considered statistically significant if P<0.05. We conducted a power analysis using G*Power [14] to determine the minimum sample size needed to detect a 1.5 fold differences in mean expression level between ccRCC patients and healthy controls, as well as between pre- and post-operative patients. 16 samples of each population were required to achieve a power of 0.85 at α = 0.05 (two-tailed test).

## Results

The serum peptidome profiles of 58 ccRCC patients, 64 healthy controls, and 20 paired pre-and post-oprerative ccRCC patients were analyzed in the current study. We evaluated changes at the peptidome level in the serum samples of ccRCC patients compared with those of healthy controls, and analyzed differences among preoperative ccRCC patients, postoperative ccRCC patients, and healthy controls. Analysis of spectra (screened from different groups) using ClinProTools version 2.2 led to the identification of distinct proteomic patterns between ccRCC and healthy controls, as well as between pre- and post-operative samples, and the identification of three potential serum proteomic biomarkers for ccRCC.

### MALDI spectrum generation and assay reproducibility

**Figure 1:**
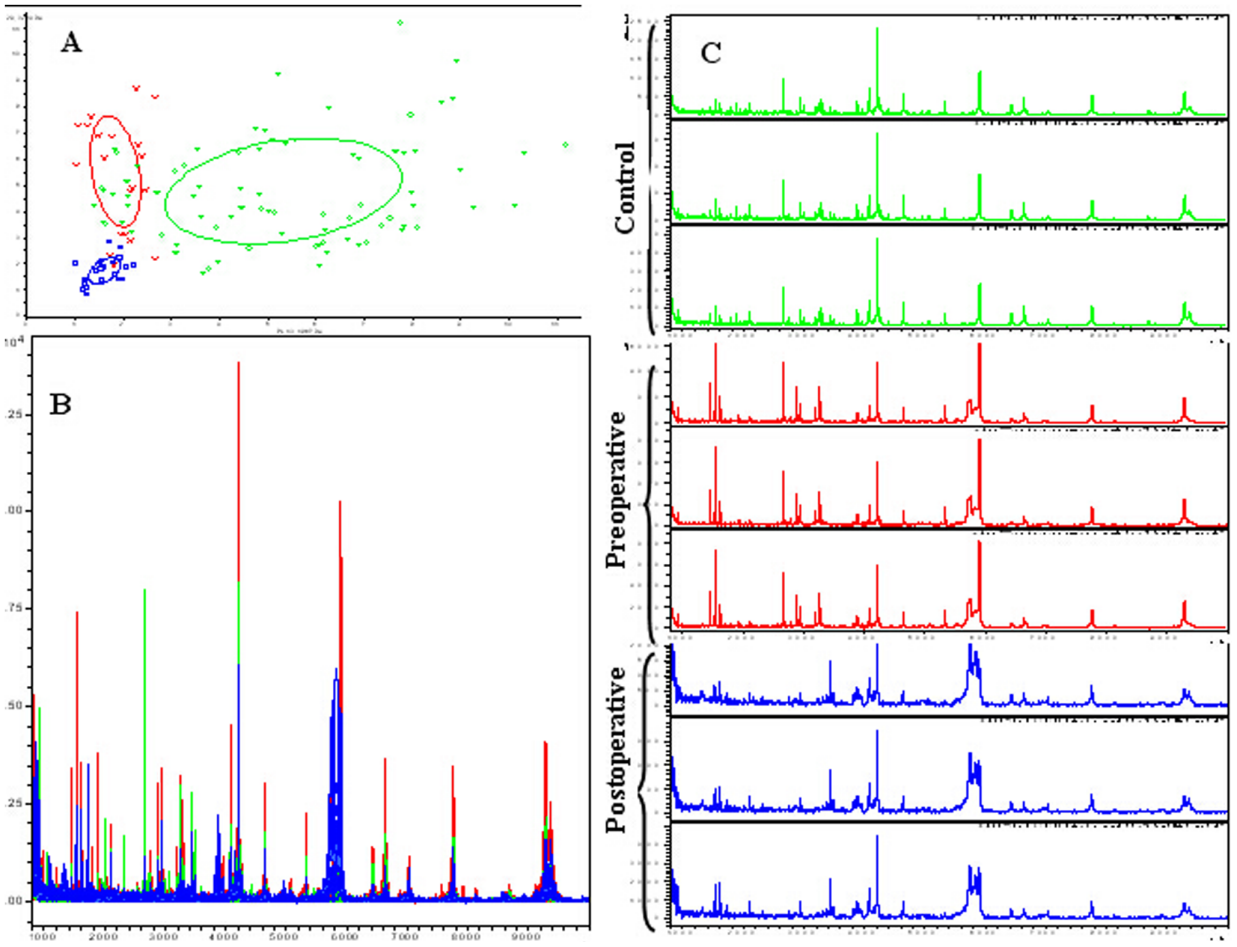
Comparative profiling of serum peptides from preoperative ccRCC patients (red), healthy controls (green), and postoperative ccRCC patients (blue). **A**. Bivariate plot of preoperative ccRCC patients (red), healthy controls (green), and postoperative ccRCC patients (blue) in the component analysis. **B**. Overall sum of the spectra in the mass range from 1000 to 10,000 Da obtained from the three groups described above. **C**. Representative mass spectra of one ccRCC patient (including preoperative (red) and postoperative (blue) serum samples) and a healthy control (green) (three spectra per sample) in the mass range from 1000 to 10,000 Da showing low variability between replicates of each sample.

Prefractionation of serum samples by MB-WCX and MALDI-TOF MS identified up to 67 peaks, of which 24 showed significantly different m/z peaks among ccRCC patients, healthy controls, pre- and post-operative ccRCC samples, with P<0.05 according to the Wilcoxon rank sum test (Table 2). In the component analysis, a bivariate plot of preoperative ccRCC patients (red), healthy controls (green), and postoperative ccRCC patients (blue) showed few overlapping regions among the three groups (Fig. 1A). Overall, the three groups showed protein profiles ranging from 1 to 10 kDa (Fig. 1B). Within this mass range, a significant number of differentially expressed proteins or peptides could be detected.

**Table 2:** Mean levels of differentially expressed proteins among ccRCC patients (n = 58), healthy controls (n = 64), pre- and post-operatives ccRCC samples (n = 20).

Mass	PTTA	ccRCC n = 58	Control n = 64	Preoperative n = 20	Postoperative n = 20
1866.63↓	<0.000001	3.13±1.25	5.72±2.63	1.98±0.56	1.73±0.36
3317.00↓	<0.000001	2.29±1.36	4.95±1.94	3.18±1.46	2.14±0.44
6433.36↓	<0.000001	2.48±1.27	4.30±2.34	2.90±1.12	1.73±0.43
1780.10↓	<0.000001	2.17±0.72	3.17±1.05	1.92±1.07	1.97±0.78
1061.34↓	<0.000001	2.69±1.03	6.19±3.28	2.05±0.92	2.27±0.86
5752.25↑	1.87E-05	2.38±1.37	1.28±0.34	2.93±2.7	5.92±3.91
5724.76↑	5.87E-05	1.75±0.82	0.77±0.19	2.22±3.23	5.67±2.27
5844.36↑	7.36E-05	2.69±1.27	1.70±0.53	2.83±1.83	7.24±3.53
5739.69↑	8.06E-05	2.07±0.98	0.94±0.22	2.49±3.31	6.50±5.06
5818.83↑	9.31E-05	2.67±1.14	1.33±0.3	2.45±1.64	6.21±3.54
**5905.23↑**	**0.00027**	**19.54±4.21**	**13.26±3.64**	**21.10±5.29**	**12.32±3.66**
**1466.98↑**	**0.00041**	**4.72±1.23**	**2.72±0.78**	**6.49±3.75**	**2.56±0.89**
4091.86↑	0.00041	9.79±3.57	6.03±2.61	9.23±3.49	5.47±1.94
1714.57↑	0.00043	7.71±3.26	1.78±0.7	5.87±1.56	7.44±3.78
**1618.22↑**	**0.0019**	**6.37±2.08**	**4.32±2.77**	**7.10±2.87**	**5.11±2.01**
1981.69↓	0.00403	1.88±0.50	5.43±2.02	1.62±0.44	1.69±0.41
1077.14↓	0.00895	2.19±1.23	3.37±2.33	2.09±1.38	2.84±0.76
1547.00↑	0.0981	9.17±4.82	5.91±3.63	10.15±3.15	5.25±2.7

Evaluation of the reproducibility and stability of the mass spectra in triplicate samples showed closely reproducible peaks (Fig. 1C). In addition, mass spectra differed among healthy controls (green), preoperative ccRCC patients (red) and postoperative serum samples (blue).

### Quality Control

To evaluate the reproducibility and stability of the mass spectra, all samples were analyzed in triplicate. The intra-assay variation of each MALDI ProteinChip assay was determined by MALDI profiling of 10 aliquots of each serum sample spotted randomly onto 10 of the 384 wells of the ProteinChip arrays along with all analytical samples. The mean value of the coefficient of variance (CV) for all 67 WCX peaks was 17.27%, with maximum and minimum values of 21.13% and 9.7%, respectively.

### Correlation of m/z peaks expression with Clinicopathological characteristics of ccRCC

Correlation between the mean expression of all detected m/z peaks and Clinicopathological characteristics of ccRCC patients were analyzed separately. While the mean expression levels of m/z peaks were found to be significantly correlated with gender difference for ccRCC (r_s_ = 0.972, p = 0.000), and it was also correlated with tumor stage (r_s_ = 0.954, p = 0.000), as well as correlate with Furhman grade (G1 vs G2: r_s_ = 0.911, p<0.000; G1 vs G3: r_s_ = 0.729, p<0.000; and G2 vs G3: r_s_ = 0.748, p<0.000, respectively).

### Peak selection and model testing

ClinProTools analysis identified up to 67 peaks, of which 18 showed significantly different m/z peaks among different groups. Of these, seven were downregulated and 11 were upregulated in preoperative ccRCC patients compared with healthy controls. Seven peptide peaks (m/z: 1061.34, 5905.23, 1466.98, 4091.86, 1618.22, 1077.14, 1547.00) showed similar values to those of healthy controls when compared with the postoperative group (Table 2), of which two (m/z: 1061.34 and 1077.14) were downregulated and five were upregulated in the ccRCC patient group.

Utilizing spectral data from ccRCC and healthy controls, three different classification models for the two groups were generated using GA, SNN and QC algorithms. These models discriminate both groups with high sensitivity and specificity. Based on the GA algorithm model, ccRCC patients could be discriminated from healthy controls with 92.71% sensitivity and 98.31% specificity. The sensitivity and specificity of the SNN model was 89.88% and 92.84% respectively, and these values were 82.55% and 83.82% for the QC model. The mean values of sensitivity and specificity of the three models were 88.38% and 91.67%, respectively.

**Figure 2:**
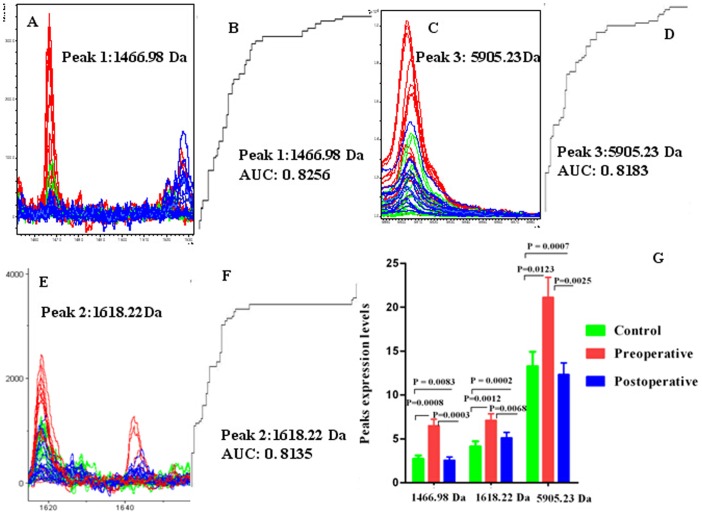
Representative spectra of three selected serum protein peaks of preoperative ccRCC patients (red), healthy controls (green), and postoperative ccRCC patients (blue). **A, C, E**. Comparison of the spectra of peaks 1466.98, 5905.23 and 1618.22 in the three groups described above. **B, D and F.** Receiver operating characteristic (ROC) curves for the three selected peaks are shown together with their area under the curve (AUC) values. **G.** Average expression levels of three selected peaks in preoperative ccRCC patients (red), healthy controls (green), and postoperative ccRCC patients (blue) and their P values. Values are expressed as the mean ± SD.

ClinProt data and the GA algorithm model showed that the preoperative ccRCC patient group could be discriminated from healthy controls and postoperative ccRCC patients with sensitivity of 91.2% and specificity of 93.6%. The five peaks used in GA model included two downregulated peaks (m/z values: 1866.63 and 1061.34) and three upregulated peaks (m/z values: 1466.98, 1618.22, and 5905.23) in the preoperative ccRCC group. The three upregulated peaks showed a tendency to return to healthy control values after surgery. Comparison of the spectra of these three peaks among preoperative ccRCC patients (red), healthy controls (green) and postoperative ccRCC patients (blue) and their receiver operating characteristic (ROC) curves were shown in Fig. 2. The area under the curve (AUC) values of these three peaks were 0.8256 (m/z: 1466.98), 0.8135 (m/z: 1618.22), and 0.8183 for m/z peaks 5905.23. (Fig. 2.)

### Identification of ccRCC serum biomarkers

**Figure 3:**
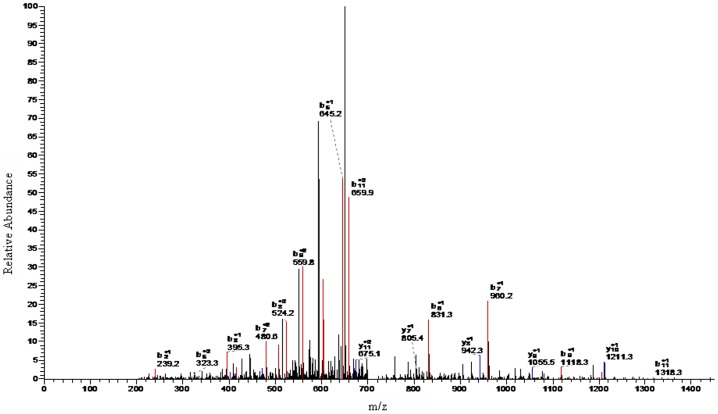
LC-ESI-MS/MS spectrum of peptides with m/z of 1466.98 Da.

**Figure 4:**
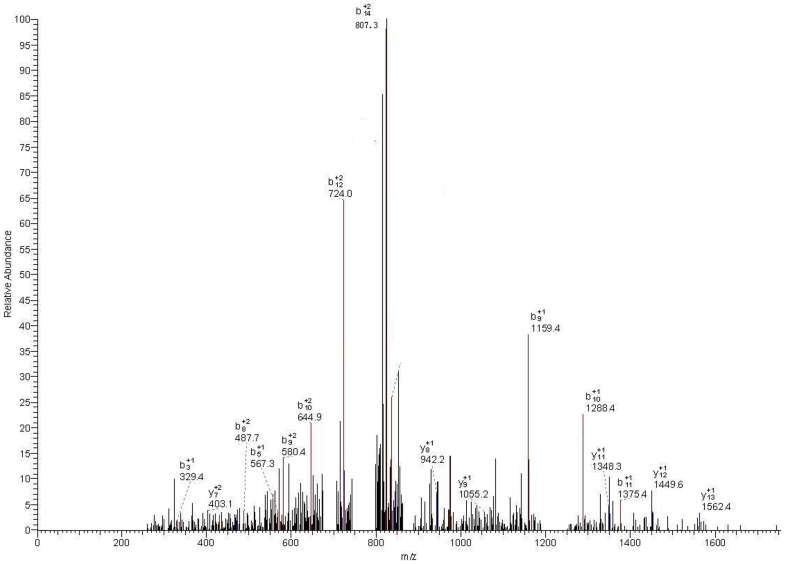
LC-ESI-MS/MS spectrum of peptides with m/z of 1618.22 Da.

**Figure 5:**
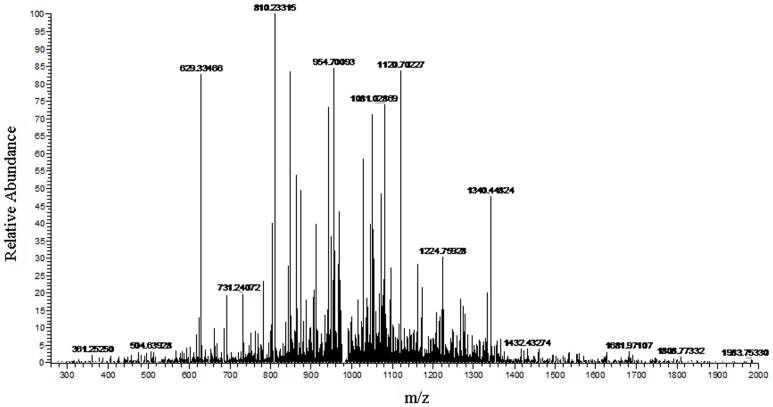
LC-ESI-MS/MS spectrum of peptides with m/z of 5905.23 Da.

A higher concentration of peptides with m/z values of 1466.98, 1618.22, and 5905.23 were evident in the spectra of the preoperative ccRCC group as compared with that of healthy controls (P<0.001), and they all showed a tendency to return to healthy control values when compared with the postoperative ccRCC group (Fig. 2). LC-ESI-MS/MS and the Mascot database (Figs. 3, 4 and 5) were used for MS/MS fragmentation of these three peptides, which resulted in the identification of the sequences KEDIDTSSKGGCVQ, AILVDLEPGTMDSVR, and IHTGENPYECSECGKAFRYSSALVRHQRIHTGEKPLNGIGMSKSSLRVTTELN. The three peptides were found to be regions of RNA-binding protein 6 (RBP6), tubulin beta chain (TUBB), and zinc finger protein 3 (ZFP3) (Table 3).

**Table 3:** Sequence identification of the three ccRCC serum protein biomarkers.

Mass m/z	Peptide sequence	International protein Index	Identity
1466.98 Da	KEDIDTSSKGGCVQ	IPI:IPI00297723.2	RBP6 (RNA-binding protein 6)
1618.22 Da	AILVDLEPGTMDSVR	IPI:IPI00011654.2	TUBB (Tubulin beta chain)
5905.23 Da	IHTGENPYECSECGKAFRYSSALVRHQRIHTGEKPLNGIGMSKSSLRVTTELN	IPI:IPI00642617.3	ZFP3 (Zinc finger protein 3)

## Discussion

The detection and diagnosis of RCC in an early stage, which remain a challenge for oncologists, could significantly reduce mortality from this cancer. In approximately 30% of RCC cases, metastatic disease is present at the time of initial diagnosis [13]. Although significant advances have been made in the treatment of RCC, few biomarkers for detection of this disease have been identified. Most current biomarker studies have relied on DNA markers [15]–[17], gene expression [18], [19] and microRNAs [20]. Nevertheless, there are no biomarkers for routine clinical use in ccRCC. Proteomic research is currently used worldwide in the search for biomarkers in different types of cancer [21], [22]. In ccRCC, Most reported proteomic studies on ccRCC or RCC were mainly from tissue specimens [23]–[28]. Noriyuki H *et al* (2013) identified nuclear N-myc downstream-regulated gene 1 as a prognostic tissue biomarker candidate in renal cell carcinoma [23]. Jones EE *et al* (2014) revealed a panel of 108 proteins that had potential disease-specific expression patterns based on MALDI imaging MS profiling of proteins in the 2–20 kDa range, from 20 matched ccRCC and distal nontumor tissues [24]. With a complex array of peptides, human serum could be value of diagnostic or prognostic markers identification [29], [30]. MALDI-TOF MS is being widely applied in the analysis of serum samples for the diagnosis of human diseases and the identification of potential biomarkers [22]. Magnetic bead-based fractionation followed by MALDI-TOF MS, combined with advanced bioinformatics (ClinProTools software) can identify biomarkers and improve the reproducibility of mass spectra, making it a valuable tool for clinical proteomic studies [21], [31]. Moreover, repeated sampling enables a better understanding of time-dependent changes that occur in response to specific stimuli, as well as information on disease progression and response to treatment [32]–[34].

In the current study, we used MB-WCX fractionation followed by MALDI-TOF MS techniques combined with ClinProTools software to analyze the serum proteomic profiles of patients with ccRCC, and generated numerous discriminating m/z peaks that could accurately distinguish cancer patients from healthy individuals, and preoperative from postoperative ccRCC patients. The ClinProTools provided predictive models for ccRCC versus healthy controls, and also between pre- and post-operative ccRCC. Moreover, the cross validation and recognition capacities of these models were 88.38% of sensitivity and 91.67% of specificity, 91.2% of sensitivity and 93.6% of specificity, respectively. We identified 18 potential biomarkers for distinguishing ccRCC patients from healthy controls. Some discriminating m/z peaks were upregulated in ccRCC patients (e.g., 1466.98, 1618.22, and 5905.23), while others were downregulated (e.g., 1061.34 and 1077.14). And three candidate peaks (1466.98, 1618.22, and 5905.23) were identified that were upregulated in the preoperative ccRCC group and had a tendency to return to healthy control values 3 days after surgery. These three peaks therefore were not only potential biomarkers for detection of ccRCC, but could also be potential markers of the response to treatment, although further studies with longer follow-up times and large cohort validation are necessary to verify these results. These three potential ccRCC serum biomarkers were identified as peptide regions of RBP6, TUBB, and ZFP3.

TUBB (beta-tubulins) is encoded by a multigene family and heterodimerize to form microtubules. Ma *et al* (2012) compared the average expression ratios of ten target reference genes in the degraded RCC samples across the time course, and the result indicated that TUBB was upregulated in malignant ccRCC tissues compared with nonmalignant renal tissues from 16 ccRCC patients [35]. This is in accordance with our finding that peptides of TUBB (m/z: 1618) were identified as being upregulated in ccRCC patients. TUBB was also reported to be associated with other cancers, and could decrease expression in uveal melanomas that subsequently metastasized compared with those that did not [36]. In cases of ovarian carcinoma, high expression levels of class III beta-tubulin appeared to be associated with earlier recurrence [37]. Several widely used anticancer drugs base their activity on β-tubulin binding, microtubule dynamics alteration, and cell division blockage [38], [39]. In addition, Zinc Finger Proteins (ZFPs) contain a conserved structural motif that mediates binding to protein, DNA and RNA [40]. Despite their abundance and important roles in human development and disease, few ZFPs have been characterized in detail [41]. RNA-binding proteins (RBPs) play a critical role in the post-transcriptional regulation of RNAs and are involved in many biological processes from embryogenesis to the regulation of cytokines in the immune system [42].

In conclusion, the results of the present study showed that MB-WCX fractionation followed by MALDI-TOF MS combined with ClinProTools software shows high sensitivity and specificity for the screening of serum proteins and could be a potential tool for the identification of patients with ccRCC. To the best of our knowledge, this is the first study to report the identification of three peptide regions (m/z values of 1466.98, 1618.22, and 5905.23) corresponding to RBP6, TUBB, and ZFP3, respectively. The method proposed in the present study could generate serum peptidome profiles of ccRCC and provide a new approach to identify potential biomarkers for diagnosis as well as prognosis of this malignancy. Our analyses are mainly limited by a limited number of cases, especially between pre- and post-operative comparsions, as well as lack of validation cohort. We will enlarge the patient cohort, and include other types of RCC as well as other renal diseases in further studies. The study is further limited by the lack of validation of the identified biomarkers as well as their functional data, for there were no corresponding peptide antibodies available for this study, although validated biomarkers are recognized as a priority in the management of patients with ccRCC [13]. The present method could also be of value for the identification of prognostic or predictive markers of response to treatment. Future studies will be aimed at developing antibodies against the three candidate markers identified and verifying their efficacy using multiple samples.
